# A yeast model for trichohepatoenteric syndrome suggests strong loss of Ski2 function in most causative mutations.

**DOI:** 10.17912/micropub.biology.000575

**Published:** 2022-05-20

**Authors:** Luisa J Orlando, Matthew K Yim, Thomson Hallmark, Michael Cotner, Sean J Johnson, Ambro van Hoof

**Affiliations:** 1 Department of Microbiology and Molecular Genetics, The University of Texas Health Science Center at Houston and The University of Texas MD Anderson Cancer Center UTHealth Graduate School of Biomedical Sciences, Houston.; 2 Department of Chemistry and Biochemistry, Utah State University, Logan, Utah

## Abstract

The intestinal and immune disorder trichohepatoenteric syndrome (THES) is characterized by mutations in human Ski2 and Ski3, also known as SKIV2L and TTC37, respectively. The mechanism by which these mutations leads to the immunodeficiency, chronic diarrhea, failure to thrive and liver disease associated with THES is unknown. To what degree THES patient mutations in Ski2 affect Ski2 function and how the differences in Ski2 function could lead to varying patient outcomes has not been studied. Here, we assayed function of THES ski2 mutants in the yeast homolog. Our results show that most THES patient mutations cause severe dysfunction in Ski2. This provides the first functional analysis of these mutations and suggests that the yeast assay may be helpful in distinguishing between pathological and benign variants.

**Figure 1. A yeast model for trichohepatoenteric syndrome suggests strong loss of Ski2 function in most causative mutations.**
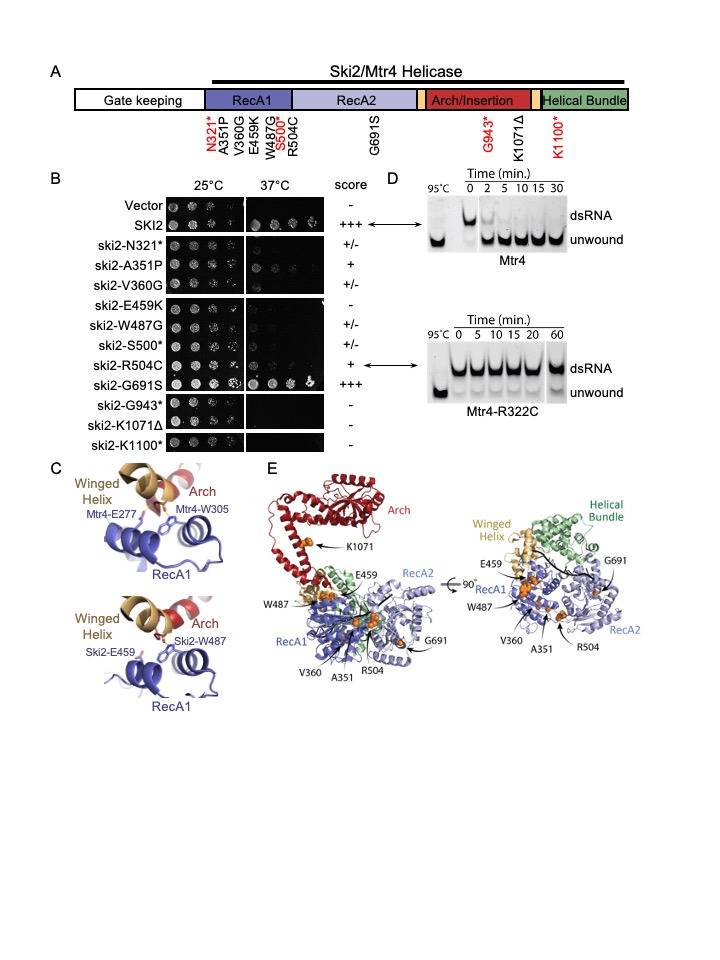
**A.** Schematic representation of Ski2 domain organization and the mutations analyzed. **B.** Representative image of growth assay of THES patient mutations in *ski2Δ dcp2-7* yeast strain. Differences in growth caused by the mutations over three replicates are qualitatively summarized in column to right. - indicates no growth even upon prolonged incubation, similar to vector control. +/- indicates some growth upon prolonged incubation. + indicates detectable growth at 3 days. +++ indicates growth similar to wild-type control. **C.** Two conserved residues that are important for Ski2 function and Mtr4 folding. **D.** RNA helicase assays of yeast Mtr4 (top) and Mtr4-R322C (corresponding to Ski2-R504C). **E.** THES patient Ski2 single amino acid mutations plotted onto the structure of Ski2, with domains colored as in panel A.

## Description

Mutations in Ski2 and Ski3 cause the intestinal and immune disorder, trichohepatoenteric syndrome (THES) by an unknown pathogenic mechanism (Bourgeois et al. 2018; Fabre et al. 2012; Hartley et al. 2010; Morton et al. 2018). The symptoms of THES vary though most, but not all, THES patient present with some degree of intractable diarrhea, inter-uterine growth retardation, woolly hair, immunodeficiency, and recurrent infections (Fabre et al. 2018; Poulton et al. 2019). Some reports suggest THES may also cause cardiac abnormalities and liver disease in some patients (Fabre et al. 2012). Why mutations in Ski2 and Ski3 only affect these specific organs/cell types is unknown as is whether specific symptoms or disease severity are linked to specific mutations.

Ski2 and Ski3 are subunits of the superkiller (SKI) complex that functions as the cytoplasmic cofactor of the RNA exosome complex (Brown et al. 2000; Halbach et al. 2013; Jacobs Anderson and Parker 1998). HBS1LV3/Ski7 physically tethers the SKI complex to the RNA exosome (Araki et al. 2001; Kowalinski et al. 2016; van Hoof et al. 2000). The SKI complex is composed of a gatekeeping module (Ski3, two copies of Ski8 and the N-terminus of Ski2) and a helicase module (the C-terminus of Ski2) (Kogel et al. 2022). Strikingly, THES-causing mutations are not distributed evenly across the SKI complex. THES-causing mutations in Ski3 are distributed evenly across the proteins and include many single amino acid changes and truncations (Bourgeois et al. 2018). THES-causing mutations in Ski8 have not been reported. Finally, THES-causing mutations in Ski2 are restricted to the helicase module and leave the gatekeeping module intact (Bourgeois et al. 2018). Specifically, homozygous single amino acid mutations that cause THES are concentrated in the RecA1 domain of the helicase module, while the most extreme homozygous truncation leaves the gatekeeping module and RecA1 domain intact (Figure 1A). Thus, the range of THES-causing mutations in Ski2 is not what would be expected if a simple loss of Ski2 function caused THES. Consistent with this, mouse studies have shown that truncating Ski2 after 42 amino acids (2 exons) is embryonic lethal (Yang et al. 2022). Limiting this truncation to only keratinocytes results in pups succumbing within 24 hours after birth (Yang et al. 2022). Therefore, this truncation does not mimic the human disease. A major limitation to understanding the molecular mechanism by which Ski2 mutations cause THES is that there is no cellular, biochemical, or model organism assay to easily assay the consequences of particular mutations. Because the RNA exosome and SKI complex are evolutionarily conserved, we use yeast as our model organism for studying effects of THES patient mutations on hSki2 function. Yeast additionally offers the possibility to test a variety of mutations relatively easily.

In yeast, mRNA degradation can occur from either the 5’ or 3’ end. Ski2, Ski3, Ski8 and Ski7 are required for 3’ to 5’ degradation of mRNAs (Araki et al. 2001; Jacobs Anderson and Parker 1998; van Hoof et al. 2000), while Dcp2 is required for 5’ to 3’ degradation (Dunckley and Parker 1999). Although inactivation of the Ski genes has a minimal phenotype, inactivation of both pathways is lethal (Dunckley and Parker 1999). We used a *ski2* deletion strain containing a temperature sensitive decapping protein, dcp2-7, to elucidate the effects of THES patient mutations in Ski2. (Figure 1B). In this strain any *SKI2* allele is viable at 25°C. However a nonfunctional *SKI2* allele is lethal at 37°C, the restrictive temperature for dcp2-7. In contrast, functional *SKI2* alleles are able to grow at 37°C even though *dcp2-7* is defective. To confirm this design, we transformed the *dcp2-7 ski2∆* strain with a wild-type *SKI2* plasmid or an empty vector. As expected, the *SKI2* plasmid allowed growth at 37°C, while the empty vector did not (Figure 1B).

Three single amino acid changes in the RecA1 domain of the helicase core have been identified in homozygous THES patients and thus most conclusively shown to be causative (E438K, W466G, R483C) ( http://umd.be/SKIV2L last accessed on 21 Feb 2022 (Bourgeois et al. 2018)). These residues are highly conserved and we analyzed the equivalent mutations in yeast ( *ski2-E459K* , *ski2-W487G* and *ski2-R504C)* . All three mutations caused a severe growth defect (Figure 1B). Other THES patients are homozygous for mutations that truncate the Ski2 protein. Of these homozygous truncating mutations, the most N-terminal one (Ser479AlafsX3 (Fabre et al. 2012)) was modeled as ski2-S500* . Two of the more C-terminal truncations (R888fs and R1063X (Fabre et al. 2012; Lee et al. 2016; Rudilla et al. 2019)) were modeled as ski2-G943* and ski2-K1100* . Both of these also caused a severe growth defect (Figure 1B). Thus, when modeled in yeast, the homozygous mutations found in THES patients cause severe defects.

We also included some mutations that have only been identified in compound heterozygous patients and thus may be THES-causing alleles, although they could also cause more severe disease in the homozygous state. A more N-terminal deletion (Q302*) was identified in a compound heterozygote patient with atypical THES (together with a later frame shift mutation (Poulton et al. 2019)). This deletion removes essentially all of the helicase module, leaving only the gatekeeping module. The corresponding mutation in yeast, ski2-N321* , also caused severe growth defects (Figure 1B). A332P and K1035∆ were also identified as compound heterozygous mutations in one THES patient (Bourgeois et al. 2018). These were modeled as *ski2-A351P* and *ski2-K1071∆* and both caused a growth defect, although the A351P strain grew better than most other THES mutant alleles (Figure 1B). An additional single amino acid change (V341G) found in compound heterozygosity with a truncation mutation (Fabre et al. 2012) was modeled as V360G and caused a severe growth defect (Figure 1B).

While in most cases we did find a growth defect for THES-causing mutation, there was one exception: G631S has been reported in three different patients as a mutation in compound heterozygosity with a truncating mutation (either R374X, not modeled in this study, or R1063X, mentioned earlier (Lee et al. 2016; Zheng et al. 2016)), but the corresponding *ski2-G691S* did not cause a major growth defect in yeast (Figure 1B). One possible explanation is that this mutation causes some defect in gene expression. For example, this mutation is in a small exon and may disrupt an exonic splicing enhancer. Alternatively, it is possible that although this Gly residue is highly conserved, changing it to Ser might have different effects in the yeast protein compared to the human protein.

As a parallel approach we attempted to determine the biochemical consequences of some THES mutations. Biochemical activity of human or yeast Ski2 requires recombinant expression of all three subunits of the SKI complex. Given that the nuclear homolog Mtr4 shows extensive sequence and structural similarity for the helicase module, we attempted to express mutant yeast Mtr4-E277K, R322C and W305G, equivalent to the three homozygous mutations most conclusively shown to be causative for THES (equivalent to human Ski2-E438K, R483C and W466G and yeast ski2-E459K, R504C, and W487G). The Mtr4-E277K and Mtr4-W305G proteins were insoluble when expressed in *E. coli* , under a variety of expression and lysis conditions. Notably, these residues both stabilize association between RecA1 and the winged helix/arch domain junction of Mtr4 and Ski2 (Figure 1C). We conclude that these mutations likely affect the overall folding of the protein, possibly disrupting packing of the helicase domains. Mtr4-R322C was successfully expressed and purified, but had strongly reduced helicase activity compared to the wild-type control enzyme (Figure 1D). This residue is positioned at the interface between RecA1 and RecA2, which may account for the loss of activity. While this manuscript was in preparation, Kogel et. al. (2022) reported that in the recombinant human SKI complex (purified using a baculovirus system) Ski2-V341G also caused a strong defect in RNA-dependent ATPase activity. Thus, the THES causing mutations include some that are likely to affect overall protein folding, and some that affect its biochemical helicase activity.

Structural models of Ski2 bound to RNA (Figure 1E) suggests that there are two clusters of single amino acid THES mutations in the RecA1 domain (A351P/V360G/R540C and E459K/W487G), suggesting that these areas of the protein are especially critical for the disease process. All of these mutations cause severe effects in our yeast modeling. This suggests that THES mutations result in defects in the 3’ to 5’ degradation of mRNA. The UMD website ( http://umd.be/SKIV2L ) reports 29 different THES-causing variants in the helicase module of Ski2. Six of these 29 variants cause a single amino acid change and we show here that five of the six cause a growth defect in yeast. The other variants either cause in frame deletions or truncations. While we have tested only a subset of these, they cause defects similar to the single amino acid changes. For many of these mutations only a single patient has been identified, making it impossible to correlate particular mutations with disease severity. It therefore remains to be determined whether the magnitude of the growth defect seen in our yeast system correlates with disease severity or the spectrum of disease features. However, our results suggest that assaying effects of mutations in the yeast homolog may be helpful in distinguishing between likely pathogenic and likely benign variants. Although it is impractical to test multiple alleles in a mouse model it would be interesting to test one disease-relevant allele in mice and compare the phenotype to that reported (Yang et al. 2022) for a truncation after 42 amino acids.

## Methods

The *ski2Δ dcp2-7* strain yAV2835 ( *mata, leu2-∆0, ura3-∆0, his3-∆1, met15-∆0, dcp2-7∷URA3, ski2∆∷KanMX* ) was created by crossing previously described *ski2∆* (Giaever et al. 2002) and *dcp2-7* strains (Wilson et al. 2007).

The wild-type *SKI2* plasmid (pAV878) was previously described (Klauer and van Hoof 2013). Mutations were introduced using QuikChange Lightning and confirmed by Sanger sequencing. Plasmids were then transformed into yAV2835 using the LiAc Transformation (Gietz et al. 1995). Colonies were selected on SC-Leu.

Strains containing wild-type *SKI2* , *ski2* mutant, or empty vector plasmids were grown from single colonies overnight, then diluted to OD 0.2 and allowed to grow to OD 0.8 - 1.2, then serial diluted 1:5 and spotted on SC-LEU plates and placed at room temp or 37°C. Each plate contained the wild-type and SKI2 controls and growth was semi-quantitatively compared to those controls over multiple days. The images in panel B were taken at day 3.

Point mutations of Mtr4 were made using a Q5 Site-Directed Mutagenesis Kit (NEB). The expression and purification of Mtr4 and Mtr4-R322C proteins were performed as described previously (Zhang et al. 2022). Both Mtr4-W305G and Mtr4-E277K constructs were insoluble using a variety of cell lines and growth conditions. Pre-steady state unwinding assays were performed as in (Taylor et al. 2014). Unwinding activity was determined by monitoring the displacement of a 16-nt RNA labeled with a fluorescein on its 5’ end, from a longer unlabeled RNA strand when incubated with Mtr4 (1200 nM) at saturating levels of ATP over time.
